# Seasonal Variation of Bioactive Alkaloid Contents in *Macleaya microcarpa* (Maxim.) Fedde

**DOI:** 10.3390/molecules16043391

**Published:** 2011-04-20

**Authors:** Kristýna Pěnčíková, Jana Urbanová, Pavel Musil, Eva Táborská, Jana Gregorová

**Affiliations:** 1 Department of Biochemistry, Faculty of Medicine, Masaryk University, Brno, Czech Republic; 2 Department of Chemistry, Faculty of Science, Masaryk University, Brno, Czech Republic; 3 Center of Medicinal Plants, Faculty of Medicine, Masaryk University, Brno, Czech Republic

**Keywords:** *Macleaya microcarpa*, isoquinoline alkaloids, HPLC

## Abstract

*Macleaya microcarpa* (Maxim.) Fedde belongs to the genus *Macleaya*, family *Papaveraceae*. Together with the better known and more frequently studied species *M. cordata* (Willd.) R. Br. it is a main source of quaternary benzo[c]phenanthridine alkaloids. Using HPLC we determined the content of eight isoquinoline alkaloids in the aerial and underground parts of 1-, 2-, 12- and 13-year old plants and followed their changes during the vegetative period*.* The dominant alkaloid of all samples collected in the end of this period was allocryptopine (3.8–13.6 mg/g for aerial parts, 24.2–48.9 mg/g for underground parts). Chelerythrine, sanguinarine and protopine were also present in both parts of the plant. Additionally, measurable concentrations of chelilutine (CL), chelirubine (CR), macarpine (MA) and sanguirubine (SR) were detected in underground parts. The most important finding was that contents of CR, CL, SR and MA in the 12- and 13-year old plant roots were significantly higher (approximately 3-fold for CR, 6-fold for CL, 5-fold for SR, and at least 14-fold for MA) than in 1- or 2-year old plants. The proportion of individual alkaloids in aerial and underground parts thus changed significantly during the vegetative period.

## 1. Introduction

*Macleaya* *microcarpa* (Maxim.) Fedde belongs, together with better known and more frequently studied species *M*. *cordata* (Willd.) R. Br. and their interspecific hybrid *Macleaya* *x* *kewensis* Turill, to the genus *Macleaya*, family *Papaveraceae*. The genus *Macleaya* is classified in the closely related genus *Bocconia* by some authors, although that genus only comprises woody plants (trees and shrubs) from tropical and subtropical areas of North and South America [1], whereas *M. microcarpa* is a perennial herb whose origin is in central China. Its appearance is very similar to *M. cordata*. The whole herb is permeated with a well-developed duct system of laticifers producing milky orange-brown latex. Stems are 0.8-1 m high, usually grey to olive green colored, glaucous, smooth, hollow, much branched in inflorescence. Alternate leaves with 4-11 cm long petiole are attractively lobed, blade glaucous abaxially and green adaxially. Flower buds are terete, flowers are small, ochre to copper colored and make up to 30 cm long soft branch panicles. Fruits are round capsules with one ovule on the base. The plant requires a moist, rich and well-drained soil, preferring half-shade [2]. 

The plant spreads through an underground rhizome which can be very invasive under optimal conditions. The herbs can reproduce by generative or vegetative reproduction. Generative reproduction is elaborate, time-consuming and less suitable for floricultural practice. Root cuttings or top buds can be used for spring vegetative reproduction. Both plants of the genus *Macleaya* are often cultivated in gardens and parks as ornamental solitary plants [3]. 

Alkaloids are considered the main bioactive constituents of both species. *M*. *cordata* was until now the more often studied species. Its aerial parts are one of the best known plant sources of the benzophenanthridine alkaloids sanguinarine and chelerythrine [4,5,6,7], and their extracts are used in agriculture and veterinary medicine [8,9,10,11,12,13]. Despite the great similarity of both species of the genus *Macleaya*, *Macleaya microcarpa* has only been sporadically studied. 

**Figure 1:**
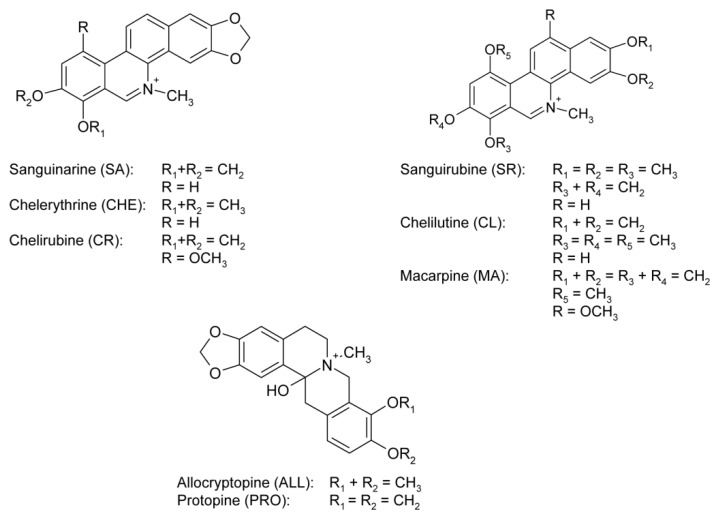
The isoquinoline alkaloids in *M. microcarpa* (all alkaloids are shown in cationic form).

One of the first comprehensive studies on *M. microcarpa* came from Slavík *et al*. [1]. They demonstrated that this plant contains protopine alkaloids – namely, allocryptopine (ALL), which is the main alkaloid of both aerial and underground parts of the plant, protopine (PRO), and a small amount of cryptopine, and quaternary benzophenanthridine alkaloids (QBAs) – sanguinarine (SA), chelerythrine (CHE), chelirubine (CR), chelilutine (CL) and the rarely occurring macarpine (MA) (Figure 1). Furthermore, trace amount of the protoberberine alkaloids coptisine and berberine and sucrose occur in roots. Aerial parts of the plant also contain a small amount of phenolic bases [1]. Recently, five new dihydrobenzophenanthridine alkaloids (maclecarpine A-E) and 10 known benzophenanthridine/dihydrobenzophenanthridine alkaloids were isolated from the roots by Deng *et al.* [14]. The presence of the alkaloids described by Slavik was not mentioned in the paper of Deng, with exception of sanguinarine and chelerythrine.

In traditional Chinese medicine, both species of the genus *Macleaya* are used mainly for treatment of some skin diseases and inflammation [14]. The extract from *M. microcarpa* displayed insecticidal and anthelmintic activity [15,16]. The roots of both plant species are often mentioned among several species that are potential good sources of the quaternary benzo[c]phenanthridine alkaloids sanguinarine and chelerythrine [17,18]. According to our previous results [17] the content of sanguinarine in *M. cordata* is lower than in *M. microcarpa*. Additionally, also indispensable amounts of the QBAs chelirubine, chelilutine, sanguirubine (SR) and macarpine were detected in the roots of *M. microcarpa*, but not in *M. cordata*. According to the current knowledge *M. microcarpa* is one of the only two known sources of the alkaloid macarpine. This alkaloid was recently reported as promising fluorescent probe for labeling of cell nuclei at fluorescence microscopy and flow cytometry [19]. While the biological effects of sanguinarine and chelerythrine have been described in many papers [20,21,22,23,24,25,26], information about biological effects of the other benzo[c]phenanthridines are rather poor; however, the contemporary results indicate that continuation in their research may provide interesting knowledge [27,28,29,30,31]. 

To expand the research on minor QBAs, it is necessary to find the best source for their isolation. As *M. microcarpa* in among their main sources, the aim of this study was to obtain more information about the production and accumulation of these alkaloids by this plant.

## 2. Results and Discussion

### 2.1. Analyses of aerial parts

We determined the content of isoquinoline alkaloids in aerial parts of 1-year, 2-year, 12- and 13- year old cultures of *M. microcarpa*. The all samples were collected at the same time, at the end of the vegetative period in October. The results are summarized in Table 1.

The dominant alkaloid in aerial parts of all samples collected in Autumn was allocryptopine; its amount varied between 3.8–13.6 mg/g, but no significant dependence on the age of plant was observable. The second main alkaloid in all samples was chelerythrine. Percentages in the Table 1 express the proportion of the given alkaloid to the total amount of four main alkaloids. It is obvious that the higher the percentage of allocryptopine is, the lower is the proportion of chelerythrine and *vice versa*, nevertheless the summed percentage of both alkaloids is nearly constant and varies between 62–69% (mean total sum of these two alkaloids was 11.06 mg/g). This might be explained by the biosynthetic relationship between the both alkaloids. Allocryptopine is the precursor of chelerythrine in the biosynthetic pathway [32]. Similar results were recorded for the protopine and sanguinarine pair that are also biosynthetically related. From this point of view, it seems that the age of the plant does not affect the final content of alkaloids in the aerial part in the end of vegetation period. Different concentrations of alkaloids in samples from various seasons are more likely consequence of variation in vegetative conditions and a stage of conversion of protopine alkaloids to benzophenanthridine alkaloids. Minor benzophenanthridine alkaloids CR and CL were also recorded in aerial parts of all samples, but only in trace amounts. Macarpine was not detected in aerial part of plants. To assess how is the content of alkaloid was affected by the vegetative period, we followed the changes of alkaloid content in the aerial parts of 13-year old plant during seven months, between April–October. The results are documented in Table 2. The proportion of individual alkaloids significantly changed during the vegetation period. The main alkaloids of the new leaves during the spring months were sanguinarine and chelerythrine whose content was more than twice higher than in autumn. During the following vegetation period the content of both benzophenanthridines gradually declined. Similar findings were published by Abizov et al [33], who compared the content of SA and CHE in aerial part of one-year old plant of *M. microcarpa* in May, July and September. 

**Table 1:** Content of isoquinoline alkaloids from aerial part of *M. microcarpa* depending on the age of herb.

Year of harvest	Age of the plant	PRO (n = 3)		ALL (n = 3)		SA (n = 3)		CHE (n = 3)	
		mg/g ± SD	%	mg/g ± SD	%	mg/g ± SD	%	mg/g ± SD	%
2008	1	1.559 ± 0.056	9.0	5.655 ± 0.239	32.6	4.370 ± 0.225	25.2	5.745 ± 0.199	33.2
2009	1	2.814 ± 0.051	18.0	6.500 ± 0.093	41.1	3.115 ± 0.107	19.7	3.371 ± 0.148	21.2
2010	1	2.610 ± 0.185	18.4	6.224 ± 0.620	43,8	2.433 ± 0.468	17.1	2.927 ± 0.239	20.7
2010	2	1.123 ± 0.053	9.8	3.815 ± 0.157	33.2	2.812 ± 0.316	22.4	3.757 ± 0.062	34.6
2009	12	2.698 ± 0.050	16.1	7.286 ± 0.140	43.5	2.961 ± 0.154	17.7	3.798 ± 0,142	22.7
2010	13	5.131 ± 0.030	20.1	13.604 ± 0.184	53.4	2.948 ± 0.212	11.6	3.678 ± 0.056	14.5

The amount of alkaloids is expressed as mass of alkaloid in 1 gram dry drug ± standard deviation. Percentage expresses the proportion of the given alkaloid on the total amount of four main alkaloids; n = number of replications.

**Table 2:** The amount of isoquinoline alkaloids during the vegetation period–aerial parts of *M. microcarpa.*

Month	PRO (n = 3)		ALL (n = 3)		SA (n = 3)		CHE (n = 3)	
	mg/g ± SD	%	mg/g ± SD	%	mg/g ± SD	%	mg/g ± SD	%
April	1.050 ± 0.070	4.2	3.040 ± 0.185	1.2	10.135 ± 1.726	40.6	10.767 ± 1.279	43.1
May	1.016 ± 0.088	4.8	2.789 ± 0.287	13.2	7.463 ± 0.855	35.3	9.877 ± 1.059	46.7
June	2.341 ± 0.083	10.0	7.225 ± 0.148	31.1	5.439 ± 0.628	23.4	8.248 ± 0.347	35.5
July	5.830 ± 0.721	16.7	18.229 ± 2.337	52.5	4.266 ± 0.512	12.3	6.384 ± 0.981	18.4
August	2.553 ± 0.098	13.4	8.134 ± 0.033	42.7	3.707 ± 0.082	19.5	4.654 ± 0.257	24.4
September	0.872 ± 0.228	3.7	14.643 ± 0.639	61.9	3.436 ± 0.159	14.5	4.700 ± 0.175	19.9
October	5.131 ± 0.030	20.1	13.604 ± 0.184	53.4	2.948 ± 0.212	11.6	3.678 ± 0.056	14.5

The amount of alkaloids is expressed as mass of alkaloid in 1 gram dry drug ± standard deviation; n = number of replications.

Oppositely, the content of protopine and allocryptopine was relatively low in the spring and culminated during July, then the content of both alkaloids fall to significantly lower values, for ALL in August and PRO in September. In October, at the beginning of vegetative quiescence, the levels of PRO and ALL in leaves partially rose. 

The aerial parts of *M. cordata* are often mentioned as the richest source of the quaternary benzophenanthridine alkaloids SA and CHE [34]. Recently Kosina *et al.* [4] studied the content of alkaloids in aerial part of *M. cordata* originating from central China and harvested in July. The content of principal alkaloids SA, CHE, ALL and PRO was in order comparable with our results for *M. microcarpa*. However, in samples of *M. cordata* the two main alkaloids were PRO and SA, while in all samples of aerial part *M. microcarpa* the two alkaloids with the highest concentration were ALL and CHE. The question remains whether this difference may be considered a chemotaxonomic feature or if it is caused by environmental conditions and other related factors. Anyway it seems that aerial part of *M. microcarpa* is as valuable a source of SA and CHE as *M. cordata*. As the content of SA and CHE is highest in the Spring and then gradually decreases, the optimal time for harvest appears to be the period when the mass of leaves is sufficient to be effective for collection and the content of alkaloids is still high. Abizov *et al*. stated the optimal period the stage between budding to the onset of flowering [33]. 

### 2.2. Analyses of roots

We next compared the content of isoquinoline alkaloids in roots of 1-year, 2-year and 12- and 13-year old cultures of *M. microcarpa* (Table 3). The all samples were collected at the same time, in October at the end of vegetative period. A similar spectrum of alkaloids was determined in roots in comparison with leaves, but the total content of alkaloids was more than twice higher. Measurable concentrations of chelilutine and chelirubine were also detected. Additionally, the presence of very rarely occurring minor QBAs macarpine and sanguirubine was confirmed. 

Allocryptopine was again the dominant alkaloid in all samples (in range 24.2–48.9 mg/g of dried drug). The amounts of protopine alkaloids, ALL and PRO, and benzophenanthridine alkaloids SA and CHE varied in samples originating from different harvests but no significant difference was observed between one-year old and perennial plant roots. However, the most important finding was that content of minor QBAs in the 12- and 13-year old plant roots is significantly higher than in one- or two-year old plants, approximately 3-fold for CR, 6-fold for CL, 5-fold for SR and at least 14-fold for MA. These alkaloids that belong to final products on the biosynthetic pathway of QBAs are likely to accumulate in older roots. 

To assess how is the content of alkaloid affected by the vegetative period, we followed the changes of alkaloid content in the roots of 13-year old plant during the seven months, between April–October. The results are documented in Table 4. The content of alkaloids in the roots changed significantly during the vegetation period and culminated during June and July for most alkaloids. The level of protopine culminated already in May, while the content of macarpine was highest during July and August. The content of alkaloids during culmination was for most of them 2–3 fold higher than in the autumn. The highest concentration of CR and CL were proved during June and July. 

**Table 3:** Content of isoquinoline alkaloids from underground part of *M. microcarpa* depending on the age of herb.

Year of harvest	Age of the plant	PRO (n = 3)	ALL (n = 3)	SA (n = 3)	SR (n = 3)	CHE (n = 3)	CR (n = 3)	CL (n = 3)	MA (n = 3)
		mg/g ± SD	mg/g ± SD	mg/g ± SD	mg/g ± SD	mg/g ± SD	mg/g ± SD	mg/g ± SD	mg/g ± SD
2008	1	11.955 ± 3.700	48.933 ± 1.572	1.791 ± 0.124	0.041 ± 0.011	2.798 ± 0.043	0.764 ± 0.102	0.179 ± 0.003	0.130 ± 0.013
2009	1	11.877 ± 3.454	44.806 ± 5.404	1.679± 0.423	0.043 ± 0.022	2.595 ± 0.013	0.764 ± 0.128	0.227 ± 0.016	0.194 ± 0.029
2010	1	12.269 ± 3.706	32.280 ± 9.697	3.028 ± 0.793	0.031 ± 0.001	4.971 ± 1.440	1.090 ± 0.284	0.181 ± 0.064	0.153 ± 0.007
2010	2	12.910 ± 0.532	41.991 ± 2.435	2.587 ± 0.107	0.047 ± 0.005	3.857 ± 0.238	1.235 ± 0.040	0.172 ± 0.003	0.134 ± 0.015
2009	12	7.567 ± 1.173	24.207 ± 0.632	1.955 ± 0.462	0.254 ± 0.005	3.027 ± 0.268	3.669 ± 0.348	1.501 ± 0.008	3.021 ± 0.328
2010	13	8.958 ± 0.348	34.947 ± 1.399	3.106 ± 0.570	0.140 ± 0.019	4.702 ± 0.469	2.720 ± 0.642	0.883 ± 0.197	1.340 ± 0.413

The amount of alkaloids is expressed as mass of alkaloid in 1 gram dry drug ± standard deviation; n = number of replications.

**Table 4:** The amount of isoquinoline alkaloids during the vegetation period—underground parts of *M. microcarpa.*

Month	PRO (n = 3)	ALL (n = 3)	SA (n = 3)	SR (n = 3)	CHE (n = 3)	CR (n = 3)	CL (n = 3)	MA (n = 3)
	mg/g ± SD	mg/g ± SD	mg/g ± SD	mg/g ± SD	mg/g ± SD	mg/g ± SD	mg/g ± SD	mg/g ± SD
April	11.843 ± 0.536	47.810 ± 2.558	2.216 ± 0.075	0.098 ± 0.009	4.573 ± 0.196	2.878 ± 0,097	0.735 ± 0.072	0.908 ± 0.153
May	12.999 ± 0.283	43.691 ± 0.235	3.268 ± 0.107	0.107 ± 0.002	6.093 ± 0.041	2.115 ± 0.041	0.650 ± 0.051	0.764 ± 0.063
June	12.686 ± 1.115	61.848 ± 4.725	4.290 ± 0.308	0.221 ± 0.036	9.854 ± 0.939	5.376 ± 0.939	1.134 ± 0.052	1.634 ± 0.263
July	11.754 ± 0.353	66.116 ± 1.421	5.583 ± 1.381	0.289 ± 0.027	15.052 ± 1.381	5.038 ± 0.155	1.783 ± 0.051	2.475 ± 0.192
August	9.256 ± 0.380	42.748 ± 1.983	2.789 ± 0.176	0.207 ± 0.016	5.600 ± 0.362	2.244 ± 0.187	1.133 ± 0.071	2.466 ± 0.053
September	6.676 ± 0.622	34.001 ± 2.477	2.941 ± 0.252	0.193 ± 0.050	6.936 ± 0.764	3.303 ± 0.723	1.520 ± 0.316	2. 311 ± 0.672
October	8.958 ± 0.348	34.947 ± 1.399	3.106 ± 0.570	0.140 ± 0.019	4.702 ± 0.469	2.720 ± 0.642	0.883 ± 0.197	1.340 ± 0.413

The amount of alkaloids is expressed as mass of alkaloid in 1 gram dry drug ± standard deviation; n = number of replications.

Chelirubine content was even higher in June than that of sanguinarine. The decrease in the concentration of all alkaloids in the roots in the end of summer can be explained as a consequence of their movement to fruits, where they probably protect the seeds against pathogens and herbivore predators [4]. Although the content of SA and CHE is also considerable in the roots, for production of SA and CHE the aerial parts are unequivocally more suitable. However, for isolation of rare minor QBAs CR, MA and CL, older roots still remain a potential useful source. 

### 2.3. Alkaloids of the plant

*M. microcarpa* have not been extensively studied, in contrast to the related spp. *M. cordata*. One of the most detailed papers comes from Slavík, who characterized the content of alkaloids on the base of isolation experiments and TLC. The spectrum of alkaloids described by Slavík *et al.* and their quantity is in good agreement with our HPLC analysis [1]. We detected allocryptopine, protopine, sanguinarine, chelerythrine as the principal alkaloids of the aerial parts and in addition to them traces of coptisine, cryptopine, chelirubine and chelilutine (Figure 1). The underground part contained protopine, allocryptopine, chelerythrine, sanguinarine, chelirubine, chelilutine, sanguirubine and macarpine. 

Deng and Hai-Lin [14] isolated five new dihydrobenzophenanthridine alkaloids and 10 known alkaloids from the roots of *M. microcarpa* but did not describe any of the protopine and benzophenanthridine alkaloids detected by us. As most of these alkaloids are dihydrosanguinarine and dihydrochelerythrine derivatives, it is a matter of discussion whether some isolated alkaloids are really native or are rather artifacts resulting from chemical interaction between an alkaloid and extraction solvent or some other component of the extract. For example, they described the presence of 6-butoxydihydrochelerythrine. We isolated this alkaloid from roots of *Dicranostigma lactucoides* HOOK.f.et THOMS (*Papaveraceae*), but only after butanol were used as the extraction solvent at slightly acidic pH [35].

## 3. Experimental

### 3.1. General

The HPLC analyses were performed using an HPLC apparatus consisting of a high pressure gradient pump LC-20AD, DAD detector SPD M20A (Shimadzu, Japan), ECOM syringe loading sample injector (ECOM, Czech Republic) with external sample loop 20 μl and C-12 column Synergi^TM^ Max-RP 80A (4μ, 150×4.60 mm ID) (Phenomenex, USA). The method was described in our previous paper [17]. In brief the extract analyses were performed using gradient elution. The mobile phase was prepared from stock solution which containing heptansulfonate sodium (0.01 mol/l) and triethylamine (0.1 mol/l) in redistilled water, pH 2.5 was adjusted by phosphoric acid. The solution A contained 25% a solution B 60% of acetonitrile (v/v). For analyses following elution profile was selected: 0–1 min. isocratic elution 20% B; 1–10 min. linear gradient from 20% to 50% B; 10–20 min. linear gradient from 50% B to 100% B; 20–30 min. isocratic elution 100% B. The flow was 0.5 mL/min and the detection was performed on DAD detector with wavelength range 180–600 nm.

### 3.2. Alkaloids and chemicals

Allocryptopine chloride, chelilutine chloride, chelirubine chloride, chelerythrine chloride, macarpine chloride, protopine chloride, sanguinarine chloride and sanguirubine chloride, all of plant origin, were isolated in the laboratory of the authors. Their identity was verified by EI-MS, ^1^H-NMR and ^13^C-NMR and their purity was not less than 98%, according to HPLC analysis. Phosphoric acid and heptanesulfonic acid were obtained from Sigma Chemical Co. (USA). Acetonitrile of HPLC grade and triethylamine were purchased from Merck (Germany). Methanol p.a. (Penta, Czech Republic) was used for extraction. 

### 3.3. Plant material and cultivated technique

*Macleaya microcarpa* (Maxim.) Fedde was cultivated in the Centre of Medicinal Plants, Faculty of Medicine, Masaryk University. The rooted rhizome cuttings (length 10-15 cm) were planted in routinely prepared soil to lines prepared before (60-70 cm from each other) and in rows 30-40 cm from each other. After planting of root cuttings watering is not necessary and reclamation is realized only. *M. microcarpa* is cold-resistant and an easily growing plant in temperate climate conditions. Three individual plants were always randomly harvested for analysis of one-year old and two-year old plants. The permanently growing plant served for collection of 12-year old and 13-year old samples in years 2009 and 2010. The plant material was wiped, dried and rough ground. Voucher specimens are deposited at the Department of Biochemistry, Faculty of Medicine.

### 3.4. Extract preparation

Ground dried drug (1 g) was extracted in methanol in a Soxhlet apparatus for 4 days. After the last extraction there was no residue after the evaporation of solvent and no peaks were detected in the extract by HPLC. Individual portions of methanol were combined and the solvent was subsequently evaporated to a total volume of 50 mL. Samples from this extract were prepared for HPLC analysis by diluting 1:9 (1 part of sample to 9 parts of stock solution).

## 4. Conclusions

*Macleaya microcarpa* (Maxim.) Fedde is a suitable source of benzophenanthridine alkaloids as well as better known and more frequently studied *M. cordata* (Willd.) R. Br., family *Papaveraceae*. The content of QBAs SA and CHE in aerial part is not significantly affected by the age of the plant. As the content of SA and CHE is highest in the Spring and then gradually decreases, the optimal time for harvest appears to be the period when the mass of leaves is sufficient to be effective for collection and the content of alkaloids is still high. A similar spectrum of alkaloids was determined in roots in comparison with leaves, but the total content of alkaloids was more than twice higher. 

The most important finding was that content of minor QBAs in the roots of 12- and 13-year old plant is significantly higher than in 1- or 2-year old plants (approximately 3-fold for CR, 6-fold for CL, 5-fold for SR and at least 14-fold for MA). Although the content of SA and CHE is also considerable in the roots, the aerial parts are unequivocally more suitable for production of SA and CHE, but older roots can be considered as a potential source for isolation of the rare minor QBAs CR, MA and CL. 
